# Emerging Roles on Immunological Effect of Indoleamine 2,3-Dioxygenase in Liver Injuries

**DOI:** 10.3389/fmed.2021.756435

**Published:** 2021-11-18

**Authors:** Lingyan Xu, Jiawei Ling, Chang Su, Yu-Wen Su, Yan Xu, Zhenzhou Jiang

**Affiliations:** ^1^Sir Run Run Hospital, Nanjing Medical University, Nanjing, China; ^2^Institute of Chinese Medicine and State Key Laboratory of Research on Bioactivities and Clinical Applications of Medicinal Plants, The Chinese University of Hong Kong, Hong Kong, China; ^3^School of Pharmacy, Nanjing Medical University, Nanjing, China; ^4^New Drug Screening Center, Jiangsu Center for Pharmacodynamics Research and Evaluation, China Pharmaceutical University, Nanjing, China

**Keywords:** IDO, liver injury, kynurenine pathway, immunoregulation, liver diseases

## Abstract

Indoleamine 2,3-dioxygenase (IDO) is one of the initial rate-limiting enzymes of the kynurenine pathway (KP), which causes immune suppression and induction of T cell anergy. It is associated with the imbalance of immune homeostasis in numerous diseases including cancer, chronic viral infection, allergy, and autoimmune diseases. Recently, IDO has extended its role to liver field. In this review, we summarize the dysregulation and potentials of IDO in the emerging field of liver injuries, as well as current challenges for IDO targets. In particular, we discuss unexpected conclusions against previous work published. IDO is induced by pro-inflammatory cytokines in liver dysfunction and exerts an immunosuppressive effect, whereas the improvement of liver injury may require consideration of multiple factors besides IDO.

## Introduction

**Figure 1:**
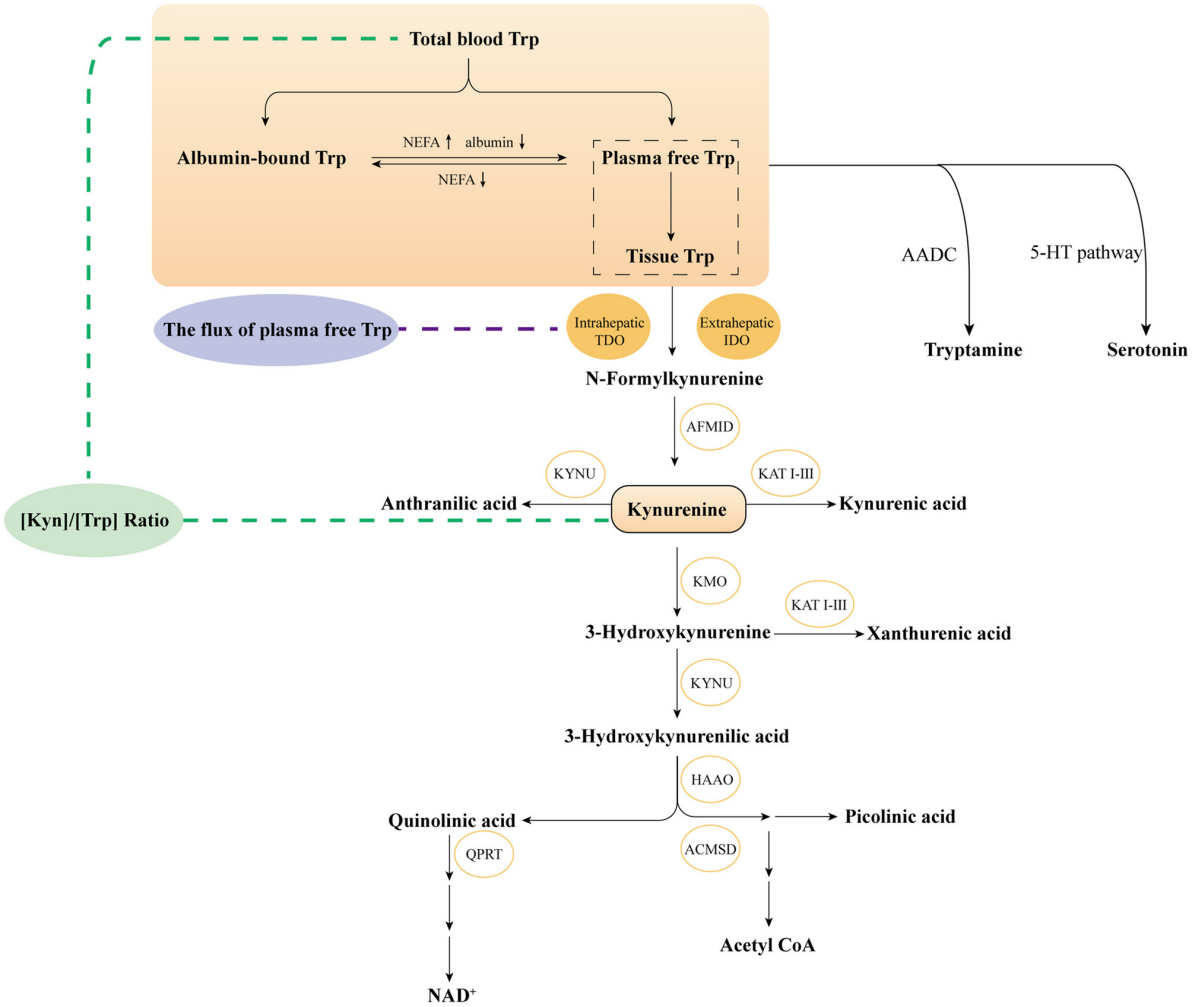
Overviewthe KP of tryptophan metabolism. Trp, tryptophan; IDO, Indoleamine 2,3-dioxygenase; TDO, tryptophan 2,3-dioxygenase; AFMID, kynurenine formamidase; KYNU, kynureninase; KAT I–III, kynurenine amino transferases I–III; KMO, kynurenine 3-monooxygenase; HAAO, 3-hydroxyanthranilate 3,4-dioxygenase; ACMSD, α-amino-β-carboxymuconate-ε- semialdehyde decarboxylase; QPRT, quinolinic acid phosphoribosyl transferase; 5-HT, 5-Hydroxytryptophan; AADC, aromatic-l-amino acid decarboxylase.

Tryptophan (Trp), as one of the nine essential amino acids, mediates energy metabolism, protein synthesis and significant bioactive molecular generation. In humans, Trp is only originated from food intake and mainly metabolized by the intestinal microbial pathway, serotonin pathway and kynurenine pathway (KP). The first two pathways consume a tiny fraction of free Trp. More than 95% of free Trp is metabolized through KP pathway to produce various key metabolites, which are extensively studied in immunology and neurology. Trp first generates N-formyl-L-kynurenine (NFK) by notable rate-limiting enzymes of Indoleamine 2,3-dioxygenase 1 (IDO1), IDO2 and tryptophan 2,3 -dioxygenase (TDO), further transforming into Kynurenine (Kyn). Kyn degradation includes three branches: (i) Kyn is metabolized into neuroactive and neurotoxic metabolites including 3-hydroxykynurenine (3-HK), 3-hydroxyanthranilic (3-HAA) and quinolinic acid (QA) by kynurenine monooxygenase (KMO) enzyme and other enzymes; (ii) Kyn directly catabolized to kynurenic acid (KA) by kynurenine aminotransferase (KAT) enzyme; (iii) Kyn further generated anthranilic (AA) by kynurenic (KYNA) enzyme (1, 2) (Figure 1).

IDO1, as the widely studied enzyme, is extensively located in various extra-hepatic cells and tissues under normal conditions but can be induced by pro-inflammatory factors such as interferon-γ (IFN-γ) during an immune response (3, 4). IDO2 shows 43% homology in amino acid sequence with IDO1 (5, 6) and located in brain, liver, thyroid and reproductive organs (6), but its mechanism is still uncertain. In this review, “IDO” hereafter refers to IDO1 or collective functional IDO enzyme activity unless otherwise specified. TDO is mainly present in the liver (3) and induced by corticosteroids, insulin and Trp (7). The most remarkable function of TDO contributes to physiological system Trp level (8). It is noteworthy that TDO has a strict substrate specificity with only L-Trp-specific, while IDO can oxidize various substrates such as L-Trp and indoleamines (7, 9). In addition, the affinity for Trp is another crucial difference. IDO shows a much higher affinity with a Km of 3–50 μM in various sources. In contrast, TDO has very high Km values, whether in rat hepatocytes (100 μM), human liver (400 μM)or the purified human enzyme (190 μM) (10). The Trp metabolism contributes to immune regulation and has been well-covered in recent reviews (1, 11–13), whereas KP imbalance is related to numerous pathologies, including autoimmunity, viral infection, central nervous system (CNS) disorders, cardiovascular and cancer.

Intriguingly, although IDO1 with a physiological state is not expressed in the liver, recent studies on liver dysfunction have found that the liver's pathological state is significantly increased (1, 2, 7, 10, 14). Iwamoto and co-workers reported that IDO mRNA and protein expression of liver and hepatocytes were not detected in the control group, while those were significantly enhanced in the acute hepatitis group, along with elevated IFN-γ and Kyn levels (15). Upregulation of IDO was consistent with previous results of HCV patients, and it was associated with IFN-γ induced by activated T cells in HCV-infected liver (16). Together, IDO dysregulation has been recorded in patients with viral hepatitis (15, 17), liver transplant (18, 19), autoimmune hepatitis (20) as well as hepatocellular carcinoma (HCC) (21, 22). Indeed, IDO expression and activity can be induced by numerous pro-inflammatory factors tumor necrosis factor-α (TNF-α), interleukin (IL)-6 (23–25), and IFN-γ (24, 26–31) and responsible for immune response. Moreover, the immune imbalance has been regarded as one of the prevailing mechanisms of liver injury (32). So, is the immunosuppressive effect of IDO related to liver dysfunction? A further investigation of IDO is urgent and available. Therefore, this review would like to discuss the functions of IDO and summarize current knowledge of IDO in liver dysfunction, along with the associated progress in therapeutically targeting IDO in each liver injury. Furthermore, the current challenges between KP and liver diseases will be especially emphasized, including the objective discussion of different conclusions to provide new strategies for future research and the development of clinical targets.

## Immunological Effects of Trp Metabolism

**Figure 2:**
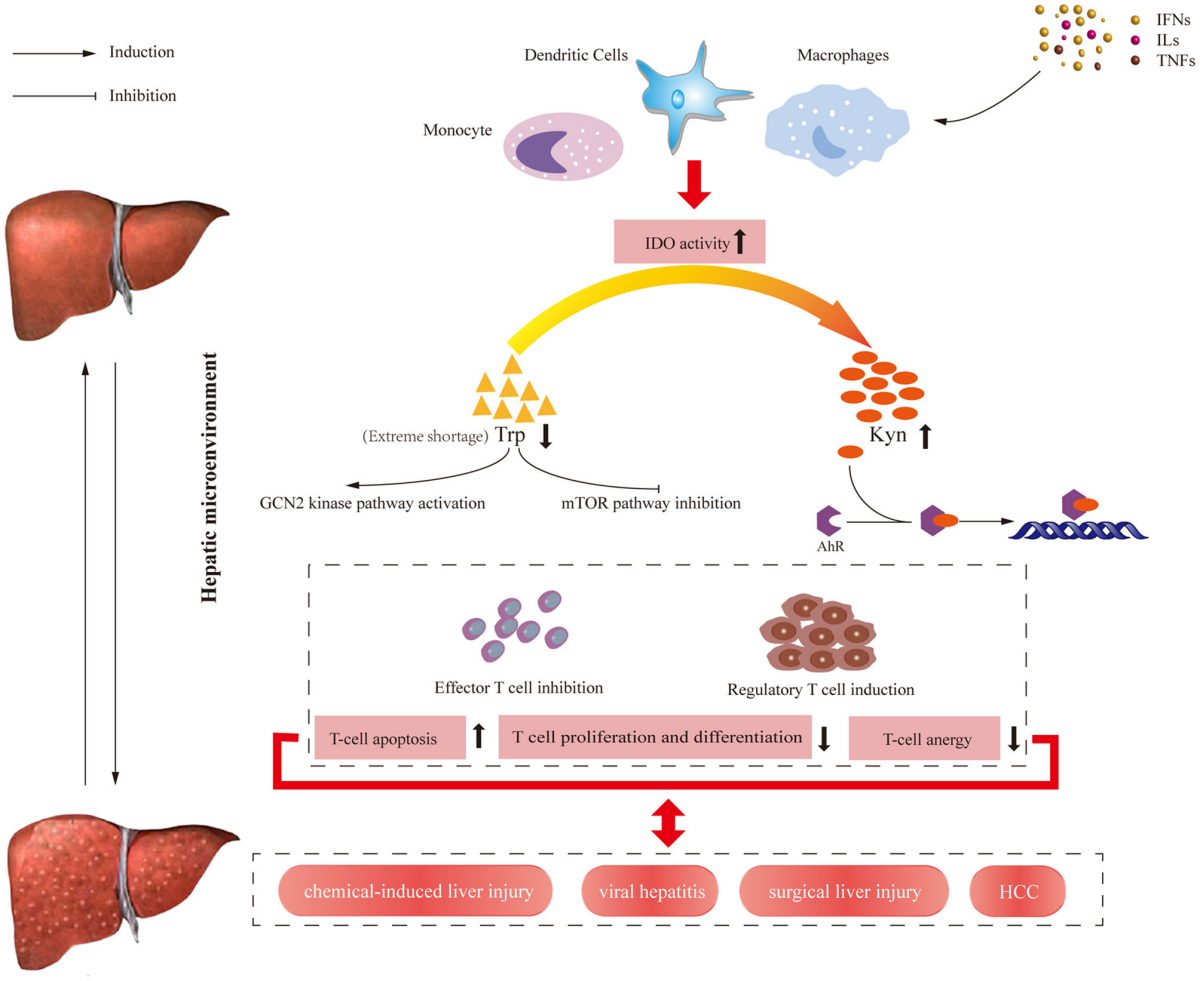
Overview of immune regulation pathways induced by IDO enzyme in humans. At the transcriptional level, IDO enzyme is expressed by various cells of the immune system and activated by cytokines and other immunomodulatory molecules such as TNF-α, IL-6, and IFN-γ. On the one hand, IDO activity results in decreasing available Trp level, which activates the GCN2 kinase pathway and inhibits the mTOR pathway, leading to the reduced number of antigen-specific T cells. On the other hand, increasing Kyn level and KP metabolite concentrations induced by IDO activate AHR pathway, leading to the increased number of regulatory T cell. The results of these signal pathways contribute to the apoptosis of effector T cells and proliferation regulatory T cells. IFNs, interferons; ILs, interleukins; TNFs, tumor necrosis factors; IDO, Indoleamine 2,3-dioxygenase; HCC, hepatocellular carcinoma; Trp, tryptophan; Kyn, kynurenine; GCN2, general control non-depressible; AHR, aryl hydrocarbon receptor; mTOR, mammalian target of rapamycin.

IDO was initially associated with establishing immune privilege and preventing T-cell-mediated allogeneic fetal rejection in mice (33). Since then, a growing line of evidence suggested IDO1 exerted crucial effects in orchestrating immune responses (34–39). It can be activated by numerous pro-inflammatory factors and T-helper cell-derived cytokines, including TNF-α, IL-6 (23–25), and IFN-γ (24, 26–31), and upregulated in different cell types including epithelial cells (23, 24, 40), macrophages (41), and dendritic cells (DCs) (42). Furthermore, enhanced IDO expression can also be modulated by inflammatory signals including transforming growth factor-α (TGF-α), nuclear factor kappa-light-chain enhancer of activated B cells (NF-κB) or transcription signal transducer and activator of transcription 3 (STAT3). These cytokines also drive liver-related inflammation and progress (32). The immunoregulatory effects mediated by IDO are mainly including as following ways: (i) Through Trp depletion, IDO inhibits the mammalian target of rapamycin (mTOR) kinase pathway and activates the General Control Non-depressible 2 (GCN2) kinase-dependent stress signaling pathway, thus inducing apoptosis and suppressing proliferation (43). However, more recent studies clarified GCN2 was activated only by extreme Trp shortage (<1 μM) (43, 44). Thus the immunoregulatory roles of Trp metabolism are mainly caused by KP metabolites rather than depletion of Trp (45). (ii) Kyn and downstream catabolites induced by IDO can activate regulatory T (Treg) cells with stimulation of the aryl hydrocarbon receptor (AHR) pathway ultimately leading to T-cell function suppression (46). (iii) IDO mediates TGF-β-driven tolerance in pDCs, which has a non-enzymic function (47). By contrast, TDO mainly maintains blood Trp homeostasis, which is dramatically upregulated when present at supraphysiological blood Trp concentrations (48, 49). More recently, TDO expression was upregulated in human tumors and exhibited a similar immunosuppressive effect to benefit tumor cells (50). Overview of immune regulation pathways induced by IDO in humans is shown in Figure 2.

## IDO and Chemical-Induced Liver Injury

### Immunological Effects of IDO in Animal Models

The inhibition of IDO can aggravate or alleviate the severity of liver damage, which depends on the immunomodulatory effect of Trp metabolism and various experimental models. An experiment on the hepatitis mice model induced by α-galactosylceramide (α-GalCer) indicated that IDO could prevent excessive immune response to weaken liver injury in this model (51). This is the first research concentrated on the effects of IDO on acute liver injury. It is worth noting that α-GalCer-induced liver injury is thought to have the potential to partially mimic autoimmune hepatitis because it is caused by the activation and apoptosis of Vα14 NKT cells. These findings indicated that the expression of IDO can down-regulate the level of the proliferation of macrophages and natural killer (NK) cells and the TNF-αproducedby these immune cells inα-GalCer-induced liver injury. The authors inferred that IDO could prevent excessive immune response in α-GalCer-induced hepatitis model based on the above results.

The CCl_4_-induced rodent models were intensively used to simulate liver fibrosis patients (52). The mechanism is primarily via trichloromethyl radicals metabolized, which destroys the biomembrane structure and triggers the inflammatory responses (53). It is reported that the liver injury in IDO-knockout (KO) mice treated with CCl_4_ was exacerbated compared with WT mice (54). In the inflammatory condition caused by the injection of CCl_4_, the authors observed IDO deficiency caused upregulation of pro-inflammatory cytokines and fibrogenic factors, contributing to the induction of activated hepatic stellate cells (HSCs) and the progression of liver fibrosis (54). Similarly, IDO inhibition with 1-D-MT after CCl_4_ injection elevated the serum alanine aminotransferase (ALT) level and increased the severity of liver injury at 16 h after disease onset (55). 1-D-MT-treated rats produced higher TNF-α levels in the liver and IL-6 levels in the serum compared with those in mock and control groups, although no differences were measured in serum MIP-2 and keratinocyte chemoattractant (KC) levels (55). Both articles pointed to IDO activity upregulated after CCl_4_ administration, while IDO inhibition exacerbated CCl_4_-induced hepatitis with enhanced cytokine and chemokines. Interestingly, it is reported by Zhong et al. that IDO deficiency attenuated CCl4-induced cirrhosis (56). In addition, a more recent article (57) observed that IDO2^−/−^ mice and administration of 1-D-MT or Kyn also prevented severe liver cell damage and liver fibrosis, which suggested that the mechanism is not attributable to the contribution of IDO1. The authors believed that Kyn produced by IDO2 in the liver might play a crucial role in CCl_4_-induced acute liver injury through a mechanism involving AHR signal transduction (57). We found that the Ogiso's group (54) and the Li's group (55) only have taken IDO into account, whereas the Hoshi's group (57) and Zhong's group (56) considered IDO2 or TDO besides IDO1. Indeed, the different endings are possibly due to a compensatory mechanism among IDO1, IDO 2 and TDO. Previous evidence suggested TDO inhibition not only increased plasma and brain Trp but also the major Trp metabolite Kyn, which suggested a compensatory mechanism by extrahepatic IDO in the absence of intrahepatic TDO (58, 59). Moreover, a study reported TDO activity was also inhibited strongly by CCl_4_ treatment (60), thus the incorporation of IDO1, IDO2 and TDO into liver dysfunction will be crucial revelation enlightenment.

Non-alcoholic steatohepatitis (NASH) is an inflamed fatty liver model subsequent to liver inflammation, fibrosis or even HCC. The animal model usually uses a high-fat diet (HFD)method. Nagano et al. reported that IDO gene silencing in the HFD-induced model aggravated hepatic inflammation and the progression of liver fibrosis. After being given pelleted HFD for 26 weeks, the IDO deficiency mice detected mixed inflammatory cell infiltration, especially T lymphocytes and macrophages in the liver. The authors speculated that IDO deficiency increased the number of lymphocytes that migrated to the liver, thereby further exacerbating liver damage (61). However, the use of HFD has been demonstrated to regulate constant brain Trp by inhibiting TDO activity, as well as NEFA was increased caused by the HFD treatment (62). Therefore, only IDO-KO treated by HFD are not free from the interference of “external” modulating factors, such as the compensatory effect of the flux of free Trp and TDO activity caused by HFD. Moreover, this article calculated the L-Kyn/L-Trp ratio as the activity of IDO enzyme, which was controversial. What is more, liver TDO activity is another determinant of the ratio (63). We found the value of Kyn/Trp ratio in HFD-mice reported by Nagano et al. was ~0.009, which was lower than a control ratio of 0.025 was reported by Ogawa et al. with the same procedure (64). It is likely contributed to the inhibition of TDO activity rather than the sole activity of IDO. Furthermore, the very low Kyn/Trp ratio in HFD mice further argues against IDO involvement. It is a pity that the authors did not report data on IDO in control mice.

Indeed, IDO deficiency does have beneficial effects in several other liver injury animal models, even though IDO expression was still increased in the model group. Using the concanavalin A (ConA)-induced liver injury mice model, Ting et al. observed that the inhibition or deficiency of IDO could alleviate murine liver damage by the reduction of inducible nitric oxide synthase (NOS) and 3-nitrotyrosine (65). As the T-cell mitotic plant lectin, ConA is recognized as a classic inducer for animal models of acute hepatitis, which can simulate the pattern of fulminant immunological liver injury (66, 67). ConA-induced hepatitis was recognized to be mainly related to ferroptosis, which was IDO-dependent. Noteworthy, *in vivo* and *in vitro* studies, IDO deficiency promoted the ferroptosis resistance by activating the expression of solute carrier family 7 member 11 (SLC7A11, also known as xCT), while reducing murine liver lesions and reactive nitrogen species (RNS) (65). In addition, another common bile duct ligation mice model also indicated IDO overexpression accelerated liver fibrosis and IDO-deficient fibrotic mice exhibited milder liver fibrosis than WT fibrotic mice by altered hepatic inflammatory cells (68). Researchers proved that IDO1 overexpression inhibited the maturation of CD11c + DCs in the liver and spleen, inhibited T cell proliferation mediated by mature DCs and worsened liver fibrosis, whereas in IDO1^−/−^ mice the above pathological phenomena were reversed (69). Similarly, in diethylnitrosamine-induced HCC, IDO overexpression and higher Kyn levels were detected in IDO-wild-type mice compared to surrounding normal tissue. Furthermore, IDO-KO mice prevented the development of HCC, which was caused by the increasing the mRNA expression levels of CD8, perforin and granzyme B (70).

Based on these previous studies (Table 1), we found that a single discussion of the contribution of IDO on the liver is obviously over simplify the problem since Trp metabolism is a considerably complex pathway in liver injury. We speculated there might be existed a compensation mechanism besides IDO in KP, which brought to light a novel view in liver injuries (78).

**Table 1:** Roles of IDO in animal models for liver injuries.

**Year**	**Model**	**IDO status**	**Study design**	**Roles of IDO**	**Effect of IDO immune modulation**	**References**
2009	HBV	+	Hepatitis B virus (HBV) transgenic (Tg) mice	Cytotoxic T lymphocytes transduction results in the upregulation of IDO, which might downregulate T-cell responsiveness	HBV infection facilitates the induction of IDO	(15)
2010	Liver transplantation	+	Rat orthotopic liver transplantations (OLT)	IDO may act as a local immunosuppressive molecule to protect transplanted cells, tissues and organs from immune attack	Protective effect against rat liver transplant rejection	(71)
2010	Attenuate liver injury	+	α-GalCer-inducedhepatitis	Decrease the number of TNF-α-producing immune cells in the liver	Protective effect against liver injury	(51)
2012	HBV	+	Hepatitis B virus (HBV)-transgenic (Tg)/IDO-knockout (KO) mice	IDO deficiency attenuated liver injury in HBV-Tg mice injected with HBV-specific CTL	Aggravated HBV	(72)
2012	Liver transplantation	+	Rat liver transplantation	The IDO level of KCs was closely associated with immune tolerance induction	Protective effect against rat liver transplant rejection	(18)
2012	Acute hepatic injury	+	CCl_4_-induced hepatitis model	IDO deficiency exacerbated liver injury in CCl_4_-induced hepatitis by inducing TNF-α and IL-6	Protective effect against liver injury	(55)
2013	Liver injury	NA	High-fat diet-induced hepatic inflammation and fibrosis	The deficiency of IDO may increase T cell activation, either directly or indirectly, by suppressing Tregs and thus contributed to a worsening of hepatic inflammation	Protective effect against hepatic fibrosis	(61)
2014	Liver injury	NA	The CCl_4_ liver-injured rats	The level of Trp increased	Biomarker	(73)
2016	Hepatic fibrosis	+	The CCl_4_-induced liver fibrosis in Mice	The deficiency of IDO aggravated the CCl_4_-Inducedliver fibrosis in Mice by suppressing the inflammatory response induced by TNF-α	Protective effect against liver injury	(54)
2016	Hepatocarcinogenesis	+	DEN-induced hepatocarcinogenesis	IDO up-regulation may contribute to the development and progression of liver carcinogenesisbyinduction of both inflammation and an immunosuppressive microenvironment	Aggravated hepatocarcinogenesis	(70)
2017	Hepatic fibrosis	UK	CCl_4_-induced liver fibrosis rat model	Trp level is changed at all time points and could be regarded as effective biomarkers for the early detection of liver fibrosis	Biomarkers	(74)
2017	Liver fibrosis	Humans,-; mice,+	CCl_4_-induced liver fibrosis mice	IDO1 deficiency attenuated CCl_4_-induced fibrosisthrough Th17 cells down-regulation andTDO compensation	Aggravated liver injury	(56)
2017	Liver fibrosis	NA	CCl_4_-induced liver fibrosis rat model	Trplevel was increased	Biomarker	(74)
2018	Liver injury	NA	ANIT-induced liver injury in rats	Trp was identified as potential biomarkers of cholestasis	Biomarkers	(75)
2020	Liver injury	UK	CCl_4_-induced acute liver injury	IDO2 deficiency attenuated CCl_4_-induced acute liver injury by AHR pathway	Aggravated liver injury	(76)
2020	Acute immune hepatitis (AIH)	+	ConA-induced AIH mice	1-MT alleviated murine liver damage with the reduction of inducible nitric oxide synthase and 3-nitrotyrosine expression alleviated murine liver damage	Aggravated liver injury	(65)
2021	Idiosyncratic drug-induced liver injury (IDILI)	UK	PD-1^−/−^mouse model of IDILI	1-D-MT decreased amodiaquine-induced liver injury in femalePD-1^−/−^mice.	The immuneresponse has many redundant feedback mechanisms that canlead to paradoxical effects	(77)
2021	BDL	+	BDL mice model	IDO1 affects the process of immune cells recruitment via inhibiting DCs maturation and subsequent T cells proliferation, resulting in the promotion of hepatic fibrosis	Aggravated liver injury	(68)

*In contrast to control group, the increase of IDO expression in model group is expressed as “+,” the decrease of that as “-.” UK, unknown; IDO, indoleamine 2,3 dioxygenase; Kyn, kynurenine; Trp, tryptophan*.

### Exploration of Therapeutic Targets for IDO

#### Insights Into Key Metabolites of KP

Studies on the metabolites of KP are widely used in various models of liver injury. By using wide-targeted metabolomics liquid chromatography-quadrupole time of flight mass spectrometry (LC-QTOF-MS) analysis, from the CCl_4_-induced liver fibrosis rat model group, urinary and serum metabolomics L-Trp increased significantly from 2 to week 8, which can be regarded as effective biomarkers for the diagnosis of hepatic fibrosis and therapeutic targets (74). These results agreed with other liver injury models showing similar findings such as α-naphthyl isothiocyanate (ANIT) induced liver injury models or non-alcoholic fatty liver disease models, L-Trp was screened for potential biomarkers of the early detection of liver fibrosis (73, 75, 76). In patients, Clària et al. reported that KP activity showed a positive correlation with overall severity of cirrhosis. Furthermore, QA and KA were the most sensitive markers of KP activation (79). Thus, KP metabolites could provide a potential biomarker in the prognosis and diagnosis of liver dysfunction (80, 81).

#### IDO Inhibitors Applied in Animal Models of Chemical-Induced Liver Injury

Danshensu, which was identified as a novel IDO1 inhibitor by molecular docking and molecular dynamics analysis, was the main biologically active ingredient isolated from an edible traditional Chinese medicinal herb called *SalviaeMiltiorrhizae Radix et Rhizoma* (Danshen). Interestingly, fibrosis reduction and inhibition of IDO1 expression and STAT3 activity were observed *in vitro* TGF-β1-induced hepatic stellate cell model and *in vivo* CCl_4_-induced rat hepatic fibrosis model after administration of Danshensu (82). Mechanistic studies indicated that Danshensu could inhibit JAK2-STAT3 signaling, which would further reduce the expression of IDO1 and downregulate the phosphorylation and nuclear localization of STAT3 (82). More importantly, overexpression of IDO1 diminished the anti-hepatic fibrosis effects of Danshensu. This study was critical preclinical data to suggest that reducing the IDO expression is beneficial to treating the liver injury. Meanwhile, another similar evidence was a characterized bioactive component isolated from the traditional Chinese medicinal herb *Panax ginseng C. A. Meyer* (Ginseng). Ginseng Rg1 also significantly reduced the aspartate transaminase (AST) and ALT expression levels in serum in CCl_4_-induced liver fibrosis in mice (wild-type and those overexpressing IDO1 by *in vivo* AAV9 vector) and HSC-T6 cells (83). IDO1 can inhibit the maturation of DCs, and Ginseng Rg1 promoted the maturation of hepatic DCs by reducing the expression level of hepatic IDO1. In addition, oral administration of Ginseng Rg1 ameliorated the deterioration of liver fibrosis induced by IDO1 overexpression and the more pronounced inhibition of DCs maturation mediated by IDO1 overexpression (83). Notably, their therapeutic effect possibly was not achieved only by inhibiting IDO due to the complexity of traditional Chinese medicinal herbs, and the TDO activity and other factors have not been reported in both studies. Almost certainly, Trp metabolism including IDO exerts an important regulatory role in liver immunity.

## Anti-Viral Infection Effect of IDO in Viral Hepatitis

### IDO Against HBV and HCV Infection by Regulating Immune Tolerance Microenvironment

Bacterial and viral infections are critical risk factors for liver injury. Epidemiological data demonstrated that chronic hepatitis C virus (HCV) and hepatitis B virus (HBV) are the most common forms of infectious liver disease. It is estimated that the total number of deaths due to liver disease caused by HBV is about 800,000, while about 500,000 deaths are caused by HCV (84). The HCV infection progression is possibly mediated by innate and adaptive immune responses in infected patients (85). Impairment of HCV-specific CD4^+^ and CD8^+^ T-cell responses (86–88) and abnormal DCs function has been observed in HCV infection (89) as well as the upregulation of IDO expression in HCV infection (25). Persistent IDO expression within the liver microenvironment may play a crucial role in reducing HCV-specific T-cell responses (6). Yang et al. (90) revealed that plasma level of IDO was associated with TGF-β expression and severity of chronic HCV infection. In the acute infection stage, the expression of IDO1 is upregulated to promote the process of anti-viral defense, as IFN-γ did so in HCV-infected liver cells to inhibit anti-HCV T cells in the chronic infection phase.

Similar to HCV, IDO overexpression was associated with HBV. The expression and activity of IDO in patients with chronic HBV infection were significantly higher than those in healthy controls (91). Further studies explicated that IDO expression was correlated with HBV viral load and responsible for immunotolerance against HBV (91). HBV infection facilitated IDO induction, mainly through response to the hepatocyte pro-inflammatory cytokine IFN-γ in mice model studies. The up-regulation of IDO was accountable for transduction of cytotoxic T lymphocytes and ultimately down-regulated responsiveness of T cells (15). Similarly, the increase of IDO expression in the livers of murine fulminant hepatitis model induced by HBV-specific cytotoxic T lymphocytes (CTLs) was also examined in another study. Moreover, IDO inhibition could reduce liver injury, with Kyn and IFN-γ cooperatively involved in the progression of liver injury (72). IDO was demonstrated as a potential and novel favorable therapeutic target for chronic HBV infection (37, 91).

### Possible Benefits of the Target IDO in Viral Hepatitis

IDO exhibited an essential role in HBV and HCV infections by constructing an immunotolerogenic microenvironment. Moreover, the prevailing theory is that the molecular mechanisms of the tolerogenic state are accompanied by chronic HBV and HCV infection, with only a weak response of CTLs against the HBV surface antigen (HBsAg). Nevertheless, another mechanism of IDO activity was discovered in a murine hepatitis model. Ito et al. immunized with a combination of α-GalCer, a specific agonist for NK inducing IDO and HBsAg on wild-type and IDO-KO mice. Only IDO-KO mice showed an increased expression of the cytokines IL-2 and IL-12b leading to the induction of HBsAg-specific CTLs (92). Besides, α-GalCer induced IDO production in CD11b^+^ cells, which inhibited the proliferation of HBsAg-specific CTLs (92). These data suggested that inhibition of IDO activity enhanced the induction of HBsAg-specific CTLs after immunization with HBsAg and α-GalCer. Thus, IDO played the role of the central mediator in the IFN-γ-induced anti-viral response since it mediated Trp depletion followed by suppressing HBV or HCV replication. Additionally, published literature has shown that the inhibitory effect of IDO1 can be used to treat chronic HCV patients, and its mechanism may be attributed to the reduced inducible nitric oxide synthase and 3-nitrotyrosine expression (65, 93).

As described above, IDO suppressed the degree of the immune response in viral hepatitis. In this case, IDO inhibition could be expected to enhance the immune response and shield mice from viral infections. Nevertheless, Duhalde Vega et al. got contrary conclusions from a hepatitis virus A59 (MHV-A59) infected mice model that 1-L-MT aggravated liver injury, and the survival rate of MHV-infected animals treated with 1-L-MT was severely decreased compared to the control group without treatment of 1-L-MT (94). Excitingly, they continued their follow-up exploration and demonstrated TDO inhibition by LM10 resulted in decrease levels of the Ab to MHV induced by the same virus infection (78, 95). The two contrary responses might be clarified by the compensation mechanism controlling Trp metabolism (96). It is confirmed by Too et al. that deletion of the IDO1 or IDO2 gene does not alter brain Trp, Kyn or the Kyn/Trp ratio, whereas TDO2 deletion increases brain Trp without altering brain Kyn (78). The above evidence indicated that there might exist compensatory effects of TDO when IDO is inhibited. Therefore, inhibition of IDO alone cannot alter the progression of liver injury.

It is known that pro-inflammatory cytokines elevated caused by viral infections can induce IDO instead of TDO. Further, another study about the contribution between IDO and TDO explained that the Trp metabolism that occurred during infection was related to several KP metabolites involved in immune response mediated by IDO rather than the amount of Trp depletion (45, 96). Thus the pro-inflammatory Kyn metabolites mediated by IDO may be the real pathogenic factor in viral infections.

Notably, it was reported in this article by Duhalde Vega et al. that liver (Trp) also increased in MHV-infected mice, instead of the expected decrease compared with that of control (95). Badawy et al. supported that the flux of plasma free (non-albumin-bound) Trp was another vital factor (63). In MHV-induced hepatitis, the plasma free Trp may be upregulated, which is supported by evidence of decreasing albumin and increasing non-esterified fatty acids (NEFA) in hepatitis patients (81, 95, 97, 98). And the increase of the plasma free Trp promotes the flux of Trp through TDO.

Based on these, once TDO activity is inhibited by LM10 treatment on MHV-infected mice, the degradation of intrahepatic Trp mediated by TDO failed and the flux of Trp through TDO is prevented, leading to the decrease of pro-inflammatory Kyn metabolites.Consequently, the flux of Trp through TDO together with IDO induction enhanced pro-inflammatory Kyn metabolites by two mechanisms in the MHV-infected mice.

Obviously, the immune response is very complicated, and the various effects on viral infection should not be simply interpreted as the sole IDO inhibition or Kyn decrease. Overall, the expression of IDO might have a significant effect on the prognosis of viral-induced patients. Trp metabolism may provide new strategies as an adjuvant therapy intervention for viral hepatitis.

## Immunological Effect of IDO in Surgical Liver Injury

### IDO Suppressing Liver Regeneration and Protecting Against Immune Rejection in Liver Transplantation

Compared with immune responses after a chemical- or viral-induced liver injury, post-hepatectomy and partial liver transplantation are also closely related to IDO activity by suppressing liver regeneration and protecting against immune rejection. This vital role of IDO in transplantation originated from a landmark study published in science magazine in 1998. Mellor et al. found that IDO suppressed the maternal T-cell response against fetus and inhibition of IDO with 1-MT caused abortion of allogeneic concepti but not syngeneic concepti (33). This work showed the most inspiring results of late years, which has completely changed the statement of IDO, from what was initially thought to be an innate defense mechanism to a potent immunomodulatory enzyme of which can down-regulate immune activation and inhibit T cell response and promote tolerance induction eventually. Since then, several studies on the effect of IDO in transplantation have been published. To determine whether IDO is the cause of liver tolerogenicity, Madeleine et al. studied IDO expression in liver-induced acceptance of kidney grafts in sensitized patients undergoing combined auxiliary liver-kidney transplantation. Combined auxiliary graft transplantation showed an increase of tryptophan degradation in peripheral blood and expression of IDO mRNA compared to regular renal transplantations (99). These results were proved by Xing et al. in a comparable rat experimental model where they demonstrated that IDO could be significantly overexpressed in rat livers after syngeneic and allograft liver transplantation compared to the sham-operated group (100). In addition, the results of immunohistochemistry showed that the number of IDO-positive cells was positively correlated with the exacerbation of rejection, and the level of inflammatory cell infiltration in the portal area was different *in vitro*, the expression of IDO gene and enzyme activity was increased in the IFN-γ-treated DC group within 7 days after transplantation compared to the untreated DC group and their survival rates were also significantly improved (100). Therefore, these results suggested that IDO might be involved in the spontaneous tolerance of liver allograft. IDO-positive DC induced by IFN-γ might relieve acute rejection and accelerate local tryptophan metabolism through IDO enzyme expression, resulting in post-liver transplantation immune tolerance (101, 102).

Besides living donor's liver transplantation, liver regeneration is another highly orchestrated process influenced by various factors. Liver regeneration is initiated immediately after the loss of hepatocytes for partial hepatectomy or partial liver transplantation, while its failure leads to hepatic injury and death. Hepatocyte proliferation is regulated by several growth factors such as TGF-α. Then hepatocytes need priming by TNF-α and IL-6, which are secreted by Kupffer cells, leading to the transition of growth factors from resting phase (G0) to interstitial phase (G1) (103, 104). Hideyuki et al. demonstrated that the liver-body weight ratio and the number of Ki-67-positive cells per field were significantly higher in IDO1-KO mice than WT mice. Likewise, the hepatic mRNA expression of cell cycle genes (cyclin D1, cyclin E) and pro-inflammatory cytokines (IL-1β, TNF-α and IL-6) were significantly higher in IDO1-KO mice than in WT mice (105). Moreover, the administration of the 1-DL-MT at the time of transplantation resulted in promoting liver regeneration (105). Therefore, these results indicated that IDO1 suppressed the production of inflammatory cytokines and subsequently inhibited hepatocyte proliferation during liver regeneration.

### Therapeutic Implications for IDO in Surgical Liver Injury

As previous studies described above, tryptophan catabolism via the IDO pathway is required for immune tolerance in allogeneic concepti rather than syngeneic concepti (33). Increasing evidence suggested that IDO might be a potential target to prevent acute rejection in spontaneously tolerant mouse liver allografts (106) and delayed rejection (107). Jerome et al. applied recombinant adeno-associated virus 2/8 (rAAV2/8) to deliver the transgene to allograft prior to transplantation (108). However, the median survival of recipients of allografts pretreated with recombinant adeno-associated virus 2/8–liver-specific promoter 1–IDO (rAAV2/8-LSP1-rIDO) vectors was 11–15 days, which was not significantly greater than that of the control group pretreated with recombinant adeno-associated virus 2/8–liver-specific promoter 1–enhanced green fluorescent protein (rAAV2/8-LSP1-eGFP) or untreated Piebald Virol Glaxo-to-Lewis liver allografts (108). These results indicated that transfecting single adenovirus-mediated IDO in rat liver allografts could not prevent acute rejection. Interestingly, Yakun et al. challenged to explore the effects of adenovirus-mediated combined genes of CTLA4Ig and IDO in the immune tolerance after orthotopic liver transplantation in rat models. Combined transfection of CTLA4Ig-IDO exhibited milder acute rejection and a higher survival rate in inducing immune tolerance after liver transplantation than the groups that using single adenovirus-mediated genes (CTLA4Ig or IDO) alone. These results were in line with previous reports indicating the therapeutic value of using DCs with IFN-γ-induced IDO expression to treat acute rejection after rat liver transplantation (100). The data exhibited a positive correlation between increased IDO-positive cells in the portal area and the severity of rejection. Meanwhile, experimental results from other *in vivo* models also pointed out that although IDO expression is upregulated in rejected grafts, merely IDO does not play a crucial role in the process of acute rejection (108–112). This result may be caused by a strong rejection process that IDO cannot reverse (113). Therefore, the combined application of IDO and other targets may become a feasible and valid method for immunological tolerance in liver transplantation and may become a promising clinical method for treating immune liver disease.

## The Immunosuppressive Role of IDO in HCC

### The Mechanisms of IDO Regulating Immune Responses in HCC

HCC is the third most common cause of cancer-associated deaths worldwide and confers a poor prognosis (114). Tumor-immune escape mechanisms have been currently proposed as emerging topics that were involved in HCC progression. Therefore, it has begun to focus on tumor immunology to reveal the immunosuppressive effect of IDO recently.

The prevailing theory suggested IDO has a tumor-promoting outcome in the occurrence and development of some solid tumors through immunosuppressive effects (115–117). IDO expression in various histologic cancer types seemed to build an immune-suppressive microenvironment (118, 119) by regulating immune cells including T effector cells (120), regulatory T cells (121), and Myeloid-derived suppressors cells (MDSC) (122, 123). In HCC pathogenesis, IDO was expressed in HCC cells after IFN-γ stimulation, which was a prognostic factor for poor survival of HCC patients (115, 124). One new subset of human CD14^+^ CTLA-4^+^ regulatory dendritic cells (CD14^+^DCs) was identified in HCC patients, which significantly suppressed T cell response *in vitro* via IDO (125). This finding added new insights into HCC induced immunosuppression mechanism related to IDO. Another study indicated that expression of PGE2 and IDO by HCC-associated fibroblasts might represent a novel mechanism by inducing NK cell dysfunction and creating favorable conditions for tumor progression (126).

Interestingly, some contradictory data demonstrated that induction of the IDO enzyme by IFN-γ exhibited opposite anticancer immune reactions in tumor-infiltrating cells of HCC. The recurrence-free survival rate of IDO-positive HCC patients was significantly higher than that of IDO-negative HCC patients, which was the first report to suggest that IDO expression at the molecular level might be essential for TIL to suppress tumor proliferation in HCC (127). Notably, IFN-γ is known to antitumor, namely a low, rather than a high IFN-γ level predicts HCC recurrence after therapy (128, 129). Indeed, the elevation of IFN-γ in HCC may reflect liver dysfunction rather than inflammation (130). Thus, besides IDO, the role of IFN-γ is not exclusive to the present experiment by Ishio et al. In addition, IDO was strongly induced in HCC cells following IFN-γ does not simply mean that IDO is expressed (strongly or otherwise) in HCC. Likewise, mRNA expression of IDO in HCC patients was performed in this article, which is also not always synonymous with IDO enzyme functional activity (129, 130). The mechanisms of primary and secondary resistant tumors were complex, including factors related to both the tumor and the host itself. Among them, immune metabolism dysregulation played an eventful role in the disease development of HCC patients.

### Therapeutic Implications of IDO for HCC

#### IDO Inhibitors

Using both subcutaneous and hepatic orthotopic models, Zachary et al. (131) found that adding IDO inhibitor1-D-MT can increase the therapeutic efficacy in resistant HCC tumors induced by high IDO. The negative results of current clinical trials for other tumors like ECHO-301 trial (132) made some researchers doubt about IDO inhibition strategy. The above discussion mentioned in this review, for different conclusions of IDO in various liver injuries, just provides some enlightenment in IDO/TDO target drug development. Indeed, new IDO inhibitors are under clinical trials, such as navoximod (NLG-919), an oral inhibitor that can inhibit IDO1 and TDO, and BMS-986205, an irreversible IDO1 inhibitor (133). Of course, we also got some excellent inspiration from off-target data of IDO inhibitors. The iper-activation of the mTOR pathway, the gut microbiota alteration, and the prolonged activation of AHR might be the main reasons for these compounds' lack of efficacy (134). In addition, these off-target effects could be expected to find new combinations and predictive biomarkers to select the most suitable crowd (135). Immune checkpoint blockade anti-CTLA-4 treatment increased IDO induction in RIL-175 tumor cells via IFN-γ (131), and similar findings were observed with anti-PD-1 therapy (131). These shreds of evidence indicated that IDO was still encouraging as an immune checkpoint inhibitor in HCC.

Based on the tumor-promoting effects of IDO and TDO, studies seeking small-molecule inhibitors for cancer treatment have been ongoing for recent years, including KHK2455, Epacadostat (INCB 024360), Indoximod, Linrodostat (BMS-986205) and Celecoxib (http://www.clinicaltrials.gov/). Ongoing results are shown in Table 2. These IDO inhibitors showed promising results in the treatment of patients with advanced malignancies. Furthermore, BMS-986205, a highly potent and highly selective IDO1 inhibitor, is currently evaluated in Phase I/II clinical trials combined with the PD-1 inhibitor nivolumab as first or second-line therapy to HCC patients (Table 2). The current research phase is recruiting, and the estimated study completion date is June 1, 2022, which is the current clinical trial of IDO inhibitors in liver cancer and has essential incentive significance for the future treatment of IDO inhibitors in the field of liver disease. In addition, another immune checkpoint blockade with anti-CTLA-4 and anti-PD-1 bifunctional antibodies has been approved for various advanced malignancies, including HCC (114, 136). The evidence above indicates that IDO inhibitors could provide better synergistic effects with other targeted immunotherapies and should prioritize clinical evaluation in HCC.

**Table 2:** List of current investigated IDO1 inhibitors. Information about the above trials can be accessed at https://www.clinicaltrials.gov/.

**IDO**	**Notes**	**Indications**	**Phase**	**Status**	**Mechanism**	**Reference**
IDO peptides	Nivolumab and PD-L1/IDO peptide vaccine	Metastatic melanoma	Phase 1 Phase 2	Recruiting	Combination therapy with nivolumab and PD-L1/IDO peptide vaccine to patients with metastatic melanoma	NCT03047928
KHK2455	Combined with avelumab	Urothelial carcinoma	Phase 1	Recruiting	KHK2455 (IDO inhibitor) plus avelumab in adult subjects with advanced bladder cancer	NCT03915405
Epacadostat	Combined with pembrolizumab	Sarcoma	Phase 2	Active, not recruiting	A study of Epacadostat, an IDO1 inhibitor, in combination with pembrolizumab in patients with metastatic and/or locally advanced sarcoma	NCT03414229
Indoximod	Combined with docetaxel	Non-small cell lung cancer Progression of non-small cell lung cancer Non-small cell lung cancer recurrent	Phase 1	Active, not recruiting	Immunotherapy combination study in advanced previously treated non-small cell lung cancer	NCT02460367
Epacadostat (INCB 024360)	Combined with CDX-1401 and poly ICLC	Fallopian tube carcinoma Ovarian carcinoma Primary peritoneal carcinoma	Phase 1 Phase 2	Active, not recruiting	DEC-205/NY-ESO-1 fusion protein CDX-1401, Poly ICLC, and IDO1 inhibitor INCB024360 in treating patients with ovarian, fallopian tube, or primary peritoneal cancer in remission	NCT02166905
Linrodostat (BMS-986205)	Combined with nivolumab	Endometrial adenocarcinoma Endometrial carcinosarcoma	Phase 2	Recruiting	Study of BMS-986205 and nivolumab in endometrial cancer or endometrial carcinosarcoma that has not responded to treatment	NCT04106414
Celecoxib 200 mg capsule	As single agent	Endometrium cancer	Phase 2	Recruiting	Neoadjuvant Celecoxib in newly diagnosed patients with endometrial carcinoma	NCT03896113
Linrodostat (BMS-986205)	Combined with relatlimab and nivolumab	Advanced cancer	Phase 1 Phase 2	Recruiting	An investigational study of immunotherapy Combinations in participants with solid cancers that are advanced or have spread	NCT03459222
Linrodostat (BMS-986205)	Combined with nivolumab and temozolomide	Glioblastoma	Phase 1	Recruiting	Nivolumab, BMS-986205, and radiation therapy with or without temozolomide in treating patients with newly diagnosed glioblastoma	NCT04047706
Epacadostat (INCB 024360)	Combined with pembrolizumab	Head and neck cancer	Phase 3	Active, not recruiting	Pembrolizumab plus Epacadostat, pembrolizumab monotherapy, and the extremeregimen in recurrent or metastatic head and neck squamous cell carcinoma (KEYNOTE-669/ ECHO-304)	NCT03358472
Epacadostat (INCB 024360)	Combined with pembrolizumab	Renal cell carcinoma (RCC)	Phase 3	Active, not recruiting	Pembrolizumab (MK-3475) plus Epacadostat vs. standard of care in mRCC (KEYNOTE-679/ ECHO-302)	NCT Number
Epacadostat (INCB 024360)	Combined with pembrolizumab	Metastatic pancreatic adenocarcinoma	Phase 2	Recruiting	Epacadostat, pembrolizumab, and CRS-207, with or without CY/GVAX pancreas in patients with metastatic pancreas cancer	NCT03260894
Indoximod	Combined with chemotherapy and radiation	Glioblastoma Medulloblastoma Ependymoma Diffuse intrinsic pontine glioma	Phase 2	Recruiting	Pediatric trial of Indoximod with chemotherapy and radiation for relapsed brain tumors or newly diagnosed diffuse intrinsic pontine glioma	NCT03006302

#### Prognostic Factors

Increasing pieces of evidence indicate that IDO inhibitors have great potential for the treatment of HCC patients. However, in fact, the immune responses among patients are widely different. This phenomenon may be closely related to the difference in IDO expression level of HCC-tumors, explaining why several immune IDO inhibitor monotherapy studies show disillusionary results such as ECHO-301 (NCT02752074). Subsequently, the high level of IDO expression was established as an important prognostic factor for the overall survival of HCC patients (115). Moreover, IDO overexpression on tumors was significantly correlated with high metastasis rates (124). Thus, IDO may be a novel favorable prognostic indicator for HCC. However, elevated levels of IDO1 are associated with poor patient prognosis in some circumstances (124, 137), but this is not always the case (127, 138). Thus, the disease-free survival was associated with the level of IDO1 expression in HCC patients. Moreover, there is a direct proportion between IDO1 expression levels and the ability of peripheral blood mononuclear cells of HCC patients to lyse HCC cell lines *in vitro* (127). These combined observations indicate that IDO1 may have great potential as a marker of prognosis in HCC patients.

Interestingly enough, IDO overexpression on tumors may have essential implications for immune-checkpoint therapy (139). It would be ideal for the prediction of patients for the degree of the immune response. The univariate analysis for overall survival (OS) showed that patients of early non-small cell lung cancer with higher levels of both genes (PD-L1/IDO-2) or (PD-L2/IDO-1) were associated with a worse OS. High levels of PD-L1/IDO-2 and PD-L2/IDO-1 co-expression have been independent negative prognostic factors (139). There are crucial implications of the features of IDO mentioned above for future immune checkpoint therapy (140).

An effective combination of immunomodulators and other treatments is another challenge for identifying predictive biomarkers (136). Recent studies indicated that combined multiple therapeutic options, such as combinations of anti-CTLA-4 with anti-PD-1/PD-L1 agents or combinations of anti-PD-1/PD-L1 agents and conventional treatments, have the most significant potential for successful treatments (141). The latest research directions are investigation in other biomarkers such as microsatellite instability, tumor mutational burden, BRAF and polybromo 1 (116, 142). These immune checkpoint inhibitors have shown unprecedented potentials based on positive results of biomarkers in multiple tumors.

## Conclusion

To date, the increasing interest in liver dysfunction has been associated with IDO enzyme. We summarize the immunomodulatory role of IDO, current treatment advances and challenges in various liver injury models including chemical-induced liver injury, viral liver injury, surgical liver injury and HCC. However, emerging contrary outcomes occurred, which attracts some arguments against the involvement of IDO enzyme in liver dysfunction even in Trp metabolism. Surprisingly, IDO is an integral part of KP in regulating liver dysfunction, which might be compensated with other factors such as TDO. The essential part of KP in the liver which is related to various KP metabolites, result in immune response and immunoregulation mediated by the activity of IDO rather than the amount of metabolic tryptophan. TDO might also be responsible for the degradation of intrahepatic Trp (45, 96). However, the relationship between “quantity” and “effect” is equally important, thus we need to consider other crucial factors, including the activity of IDO, TDO, Kyn monooxygenase and kynureninase, the flux of plasma free (non-albumin-bound), Trp through TDO, the Kyn/Trp ratio, the level of KP metabolites and any other factor that alters plasma Kyn disposition (63). In addition, we gave a more rational appraisal against some aspects of present IDO research, such as the somewhat indiscriminate use of the ratio in plasma or serum of concentration of Kyn to that of Trp, i.e., the (Kyn)/(Trp) ratio, to express IDO activity. Thus, it may be more informative to assess the IDO or TDO status by other means, i.e., mRNA expression and enzymatic activity. It is good enlightenment for future studies on the mechanism of KP in liver dysfunction, even other diseases. For instance, this review may provide some new inspired movements of the expected promising IDO1 inhibitors in initial studies but terminated in recent phase III trial. Dual IDO and TDO inhibitors or effective combination of other immunomodulators may also have great prospects.

## Author Contributions

LX and JL reviewed the literature and wrote the manuscript. CS, YX, and Y-WS reviewed and revised the manuscript. ZJ reviewed the literature and contributed to the conceptualization of the manuscript. All authors contributed to the article and approved the submitted version.

## Funding

The work was supported by the National Natural Science Foundation of China (82074114) and Nanjing Medical University Fund (NMUB2020320).

## Conflict of Interest

The authors declare that the research was conducted in the absence of any commercial or financial relationships.

## Publisher's Note

All claims expressed in this article are solely those of the authors and do not necessarily represent those of their affiliated organizations, or those of the publisher, the editors and the reviewers. Any product that may be evaluated in this article, or claim that may be made by its manufacturer, is not guaranteed or endorsed by the publisher.

## Abbreviations

IDO: Indoleamine 2,3-dioxygenase
HCC: hepatocellular carcinoma
KP: kynurenine pathway
Trp: tryptophan
DCs: dendritic cells
TNF-α: tumor necrosis factor-α
IL-6: Interleukin-6
TGF-α: transforming growth factor-α
α-GalCer: α-galactosylceramide
1-L-MT: Levo-1-methyl tryptophan
1-D-MT: Dextro-1-methyl tryptophan
CCl_4_: carbon tetrachloride
HFD: high-fat diet
Kyn: kynurenine
AHR: aryl hydrocarbon receptor
HCV: hepatitis C virus
HBV: hepatitis B virus
CTLs: cytotoxic T lymphocytes
mTOR: mammalian target of rapamycin
STAT3: signal transducer and activator of transcription 3.
